# TLR2/4 deficiency prevents oxygen-induced vascular degeneration and promotes revascularization by downregulating IL-17 in the retina

**DOI:** 10.1038/srep27739

**Published:** 2016-06-14

**Authors:** Chang He, Peilong Lai, Jing Wang, Tian Zhou, Zijing Huang, Lingli Zhou, Xialin Liu

**Affiliations:** 1 State Key Laboratory of Ophthalmology, Zhongshan Ophthalmic Center, Sun Yat-Sen University, Guangzhou 510060, P. R. China; 2 Department of Haematology, Guangdong General Hospital, Guangdong Academy of Medical Sciences, Guangzhou 510080, P. R. China

## Abstract

Vascular degeneration is a critical pathological process in many human degenerative diseases, which need efficient ways to revascularization. However, little is known about cellular and molecular mechanisms that are used during vascular degeneration and revascularization. Here, we show that Toll-like receptor 2 and 4 (TLR2/4) double deficiency suppressed hyperoxia induced retinal vessel regression in an oxygen-induced retinopathy (OIR) model. Notably, the TLR2/4−/− mice experienced more revascularization after reduced vessel regression compared with wild-type mice, accompanied with less activation of glial cells. Mechanistically, TLR2/4 activation can tip the balance between Th17 cells and regulatory T cells towards Th17 cells, a critical source of the IL-17A. Less migration and infiltration of IL-17A-expressing proinflammatory cells but elevated regulatory T cells were observed in OIR-retinae from TLR2/4−/− mice. Coincidentally, TLR2/4 deficiency suppressed IL-17A production and increased expressions of anti-inflammatory genes. Furthermore, IL-17A promoted activation of glial cells. IL-17A blockade using a neutralizing antibody alleviated retinal cell apoptosis and glial activation in C57/B6-OIR mice, demonstrating the important role of IL-17A pathway in glial function during revascularization. Thus TLR2/4-mediated IL-17A inflammatory signaling is involved in vessel degeneration and revascularization, indicating that modulation of the TLR2/4-IL-17A pathway may be a novel therapeutic strategy for degenerative diseases.

Vascular degeneration is a critical pathological process in numerous degenerative diseases^1^,^2^, such as stroke, myocardial infarction, retintis pigmentosa, and so on. Revascularization is a valuable treatment for vessel regression and degeneration and needs to be tightly regulated^3^. Inflammation contributes to vascular degeneration and retard the normal revascularization of the ischemic retina^4^,^5^. However, the detailed cellular and molecular mechanisms of the inflammation during this process are unclear.

Toll-like receptors (TLRs) activation triggers inflammation after recognizing pathogens or endogenous danger signals, which could play an important role in vascular diseases^6^. TLR activation in immune cells has been investigated extensively. Recently, an increasing number of studies have identified an essential role for TLRs on nonmyeloid cells, such as vascular and neural cells^7^,^8^. Among the 13 mammalian TLRs, TLR2 and TLR4 show powerful ability to promote an angiogenic response that is mediated by the direct stimulation of endothelial cells (ECs)^9^,^10^. For example, TLR4-mediated inflammation is responsible for retinal angiogenesis^6^. However, the exact role of TLR2 and TLR4 in vascular degeneration and revascularization remains unknown. The oxygen-induced retinopathy (OIR) mouse is a useful model to study serial vascular processes, including vascular degeneration and intraretinal revascularization^11^,^12^. Here, we used the OIR model to investigate the role of TLR-mediated inflammation in vascular degeneration and revascularization process.

During retinal vascular development, new vessels extend and spread following the guidance of retinal astrocytes^13^. In the OIR model, retinal glia are significantly activated in the ischemic retina^6^,^14^. Excessive activation can induce significant gliosis and impair tissue function^15^, including retinal vascular injury. TLR2 and TLR4 are abundantly expressed on glial cells including astrocytes, Müller, oligodendrocytes, and Schwann cells, suggesting a putative inflammatory role of TLR2/4 on glial function^16^,^17^. In hypoxic retinae, the role of TLR2 and TLR4 on glia activation and their effect on retinal vasculature needs to be elucidated.

In response to TLR activation, many inflammatory cytokines are expressed and cascaded. Among them, interleukin 17A (IL-17A) is an important pleiotropic cytokine that can induce inflammation and an autoimmune response and has a profound effect on angiogenesis^18^,^19^. IL-17A can promote neovascularization by stimulating ECs migration and regulating the production of a variety of proangiogenic factors^20^. However, several reports suggested that IL-17A could inhibit tumor development and neovascularization^21^. The exact role of IL-17A -mediated signaling in vascular degeneration and revascularization in OIR is still unclear.

In this study, we evaluated vascular regression and revascularization in TLR2/4 double knockout mice using the OIR model and further explored the cellular and molecular mechanisms. We also explored retinal glial activation during the process of vascular regression and revascularization. Furthermore, the role of TLR2/4 activation in the production of pro-inflammatory IL-17A and the effect of IL-17A on retinal glial function was deeply investigated. The results revealed that TLR2/4-mediated IL-17A inflammation contributes to vessel regression and impairs revascularization.

## Results

### TLR2/4 deficiency suppressed vessel regression and facilitated retinal revascularization in an OIR model

**Figure 1:**
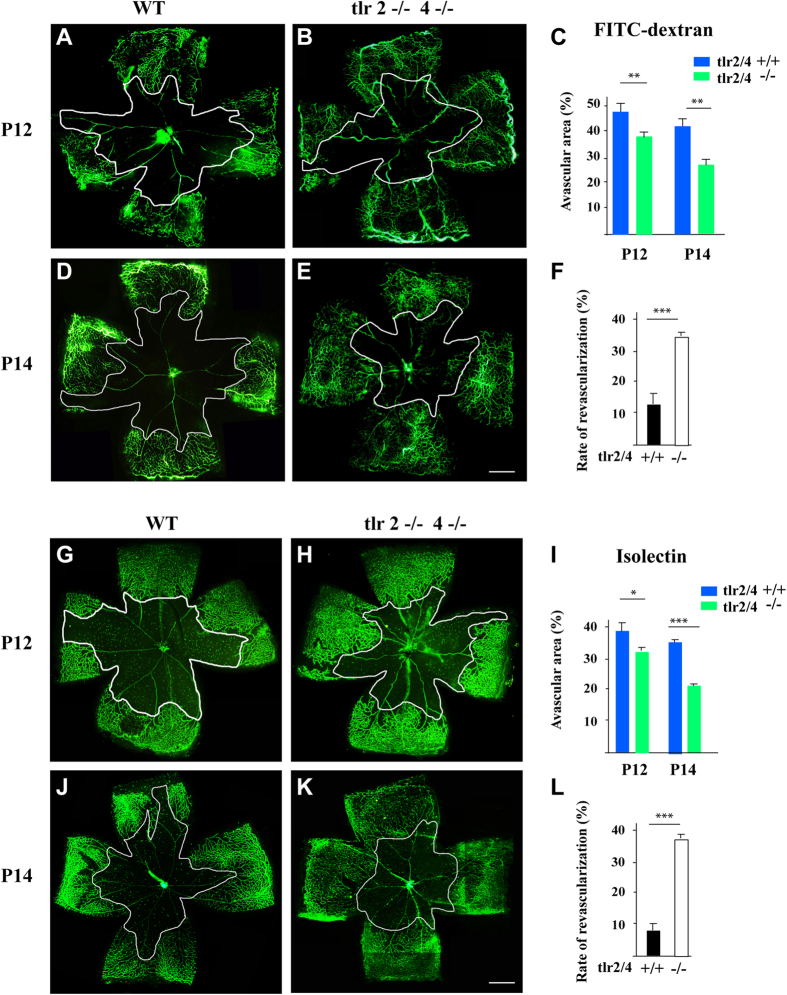
TLR2/4 contributed to vessel regression and inhibited retinal revascularization in an OIR model. **(A–C)** FITC-dextran perfusion revealed central vaso-obliteration after five days of hyperoxia (P12) in WT mice. The avascular region was reduced in TLR2/4 deficient mice compared to WT mice. **(D–F)** Similarly, TLR2/4-deficient mice showed a less central avascular area at P14. The quantified results revealed that the rate of revascularization, calculated as the difference in the avascular area from P12 to P14 divided by the avascular area at P12, is significantly increased in TLR2/4 knockout mice. **(G–L)** Isolectin B4 staining in whole-mounts also revealed a more severe vessel regression with larger avascular areas in WT retinae than TLR2/4 KO retinae at P12. In addition, TLR2/4 deficiency improved revascularization by significantly reducing the avascular area from P12 to P14. Scale bar: 300 μm. n = 8, *p < 0.05, **p < 0.01, ***p < 0.001.

Simultaneous activation of different TLRs can exert synergistic effects^22^,^23^. The synergistic role of TLR2 and TLR4 in neurovascular diseases has been investigated^24^,^25^. In this study, we used TLR2 and TLR4 double knockout mice to investigate their role in vessel regression and degeneration, which is the vital pathological change in numerous degenerative diseases. The oxygen-induced retinal vessel regression model was established. The pups at P7 were exposed to 75% oxygen to induce the vascular degeneration for up to 5 days (until P12) and then allowed to recover and began revascularization in room air (relative hypoxia condition) for additional days. The retinal whole-mount after systemic infusion of FITC-dextran was used to visualize the non-perfusion vessels and to assess vessel regression at P12. As shown in Fig. 1A–C, all hyperoxia-exposed mice exhibited central vaso-obliteration at P12. However, TLR2/4 deficiency reduced the areas of avascular regions compared to WT mice. Furthermore, the ensuing relative hypoxia began to induce revascularization with a less central avascular area and more capillary beds. At P14, the first stage of revascularization, TLR2/4 deficiency facilitated revascularization in the ischemic central capillary beds with an increased rate of revascularization, which was calculated as the difference in the avascular area from P12 to P14 divided by the avascular area at P12 (Fig. 1D–F). To further corroborate such observations using a systemic infusion of FITC-dextran, we also performed isolectin B4 staining in whole-mounts to further detect the role of TLR2/4 in the vascular profile of the OIR model. As expected, IB4 staining also revealed more severe vessel regression with larger avascular areas in the WT retinae compared to that in TLR2/4 KO mice at P12 (Fig. 1G–I). The revascularization with a decreased avascular area from P12 to P14 was more obvious in TLR2/4 KO mice than in WT mice (Fig. 1J–L). Taken together, these data suggest that TLR2/4 deficiency reduced the vessel regression and facilitated retinal revascularization in the OIR model.

### TLR2/4 deficiency relieved glia activation during retinal revascularization

**Figure 2:**
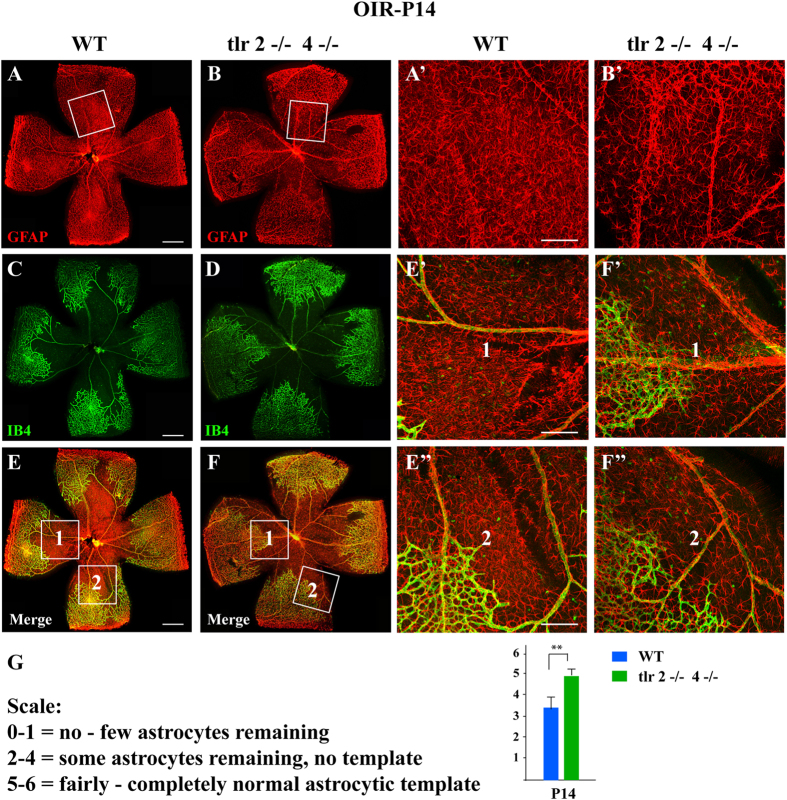
TLR2/4 accelerated glia activation during retinal revascularization. **(A**,**B)** GFAP staining in the retinal whole mounts showed the activation of glial cells in the general view. **(C**,**D)** IB4 staining results revealed reduced retinal vaso-obliteration areas in TLR2/4 knockout mice. **(E**,**F)** Merged images of both GFAP and IB4 staining. **(A’**,**B’)** Magnified view of the framed area in A-B showed that star-shaped GFAP astrocytes labeling increased while GFAP spotted staining of Müller cell endfeet significantly decreased in TLR2/4 KO mice compared with WT mice. **(E’,E”,F’,F”)** Magnified view of framed area 1 and 2 in E-F revealed that the activation of Müller cells with spotted staining of cell endfeet was greater in the avascular adjacent region from WT mice compared to TLR2/4 KO mice. **(G)** Scale 0–6 was applied to quantify the degree of astrocyte template. Statistics revealed a significant increase in astrocyte scores in the TLR2/4 KO mice at P14. Scale bar: 300 μm in (**A–F**), 100 μm in (**A’–F”**). n = 8, **p < 0.01.

Reactive retinal glial cells, including astrocytes and Müller cells, play a central role in retinal revascularization after ischemic injury^26^. We therefor assessed the activation and survival of retinal astrocytes and Müller cells in OIR model from WT and TLR2/4 double knockout mice. Retinal glial activation is often exhibited as disorganized and over-proliferated Müller endfeet as well as destroyed astrocytes templates. As showed in Fig. 2A,C,E, there was a profound loss of astrocytes in the retinal vaso-obliteration areas from the WT-OIR mice at P14, as indicated by the absence of star-shaped GFAP astrocyte labeling and an increase in Müller cell endfeet GFAP reactivity, which is a hallmark of retinal disease. The magnified images (Fig. 2A’,E’,E”) of the vaso-obliterated zone in A and E revealed obviously the activation of Müller cells presented as densely GFAP spotted staining of the cell endfeet. In contrast, in retinas from TLR2/4 knockout mice, the astrocytes presented with an almost complete GFAP-stained star-shaped template, but not with GFAP spotted positive staining of reactivated Müller cells (Fig. 2B,D,F), indicating that TLR2/4 deficiency inhibited reactive gliosis (Fig. 2B’,F’,F”). When quantified as scores from 0 to 6, with greater scores representing a more normal astrocytic template, there was a significant increase in astrocyte scores in TLR2/4 KO mice during revascularization after vessel regression (Fig. 2G). Taken together, these results indicated that TLR2/4 deficiency inhibited the loss of astrocytes and activation of Müller cells in retinal revascularization after vessel regression.

### TLR2/4 deficiency suppressed infiltration of IL-17A-expressing inflammatory cells into the retina

**Figure 3:**
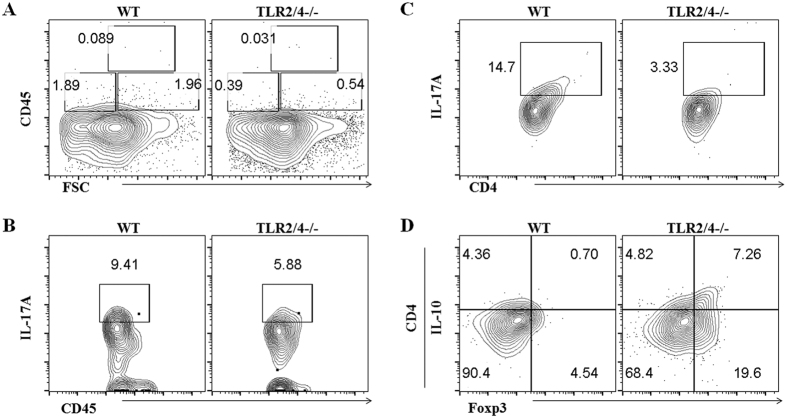
TLR2/4 promoted the infiltration of IL17A-expressing inflammatory cells in retina of the OIR model. **(A)** Flow cytometric analysis of CD45^+^ leukocyte cells in the retina on day 14 in the OIR model. Significantly more CD45^+^ leukocyte infiltrated in retinae from WT-OIR than those of TLR2/4−/− -OIR. **(B)** The IL-17A-expressing CD45^+^ leukocytes were reduced significantly in retinae of TLR2/4−/− OIR compared with WT-OIR ones. **(C)** The classic IL-17^+^CD4^+^ Th17 cells were also observed decreased in retinae of TLR2/4−/− OIR. **(D)** In contrast, the IL-10-expressing CD4^+^Foxp3^+^ Treg cells were elevated markedly.

It is of note that TLR2/4 is required to induce retinal inflammation. We therefor examined whether TLR2/4 signaling triggered IL-17A-mediated inflammation in OIR model by flow cytometry. Overall we found a substantial reduction in the level of CD45^+^ leukocyte in the TLR2/4−/− -OIR compared with WT-OIR (Fig. 3A), indicating less inflammation in TLR2/4 deficient mice. To further understand the type of inflammatory cells, we examined the expression of IL-17A, a critical pro-inflammatory cytokine that is linked to the pathogenesis of diverse autoimmune and inflammatory diseases, on these cells. The flow cytometry results showed that the IL-17A expression in all CD45^+^ leukocytes (Fig. 3B) as well as CD4^+^ T cells (Fig. 3C) were decreased significantly in retinae of TLR2/4−/− OIR compared with WT ones, indicating not only classic Th17 cells, but also other leukocytes like neutrophils, monocytes, γδT cells, and natural killer T cells, were involved in TLR2/4-induced retinal inflammation. In contrast, the IL-10-expressing Foxp3^+^ Treg cells were elevated markedly (Fig. 3D). These data suggested that TLR2/4 deficiency weakened the infiltration of CD45^+^ inflammatory cells, especially the IL-17-expressing cells whereas increase anti-inflammatory IL-10-expressing cells.

### TLR2/4 inhibited the Th17-skewed T cell response, with a greater expansion of Tregs in the OIR model

**Figure 4:**
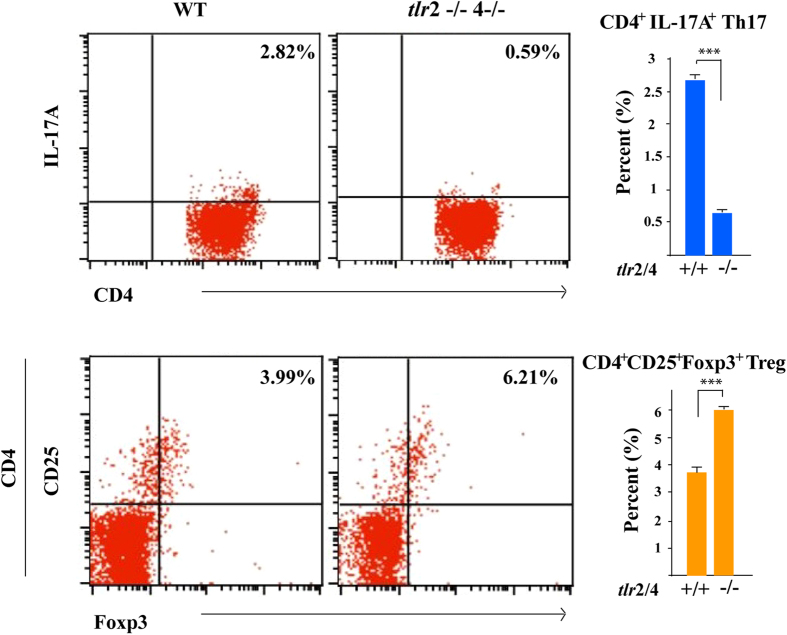
TLR2/4 promoted the Th17-skewed T cell response in the OIR model. Flow cytometric analysis of CD4^+^ T cells in the periphery on day 14 in the OIR model. The lymphocytes were isolated from the spleen and lymph nodes. The numbers in the quadrants indicate the percentage of CD4^+^IL-17A^+^ and CD4^+^CD25^+^Foxp3^+^ cells in the peripheral lymphoid organs. The results showed decreased IL-17A-expressing Th17 cells, but more CD4^+^CD25^+^Foxp3^+^ expression in Treg cells in TLR2/4-KO-OIR compared to WT-OIR mice. n = 6, ***p < 0.001.

IL-17A is mainly produced and secreted by Th17 cells and TLRs on antigen-presenting cells are crucial for CD4^+^ T cell differentiation towards Th17 cells^27^. CD4^+^ T cells differentiation in the peripheral immune system could indirectly present the immune condition in inflamed local tissue like OIR-retina. Therefore the frequencies of the Th17 cells and the anti-inflammatory Tregs in the peripheral lymphoid organs of OIR models were also analyzed. As shown in Fig. 4, the frequency of CD4^+^IL-17^+^ Th17 cells in the TLR2/4−/− -OIR model was markedly reduced compared to the WT-OIR model. In contrast, the significantly increased frequency of CD4^+^CD25^+^Foxp3^+^ Treg cells was observed in the TLR2/4−/− -OIR model, suggesting that TLR2/4 accelerated the generation of IL-17A-producing Th17 cells and impaired Tregs in the peripheral lymphoid organs of OIR model.

### TLR2/4 promoted the expression of IL-17A in the retina

**Figure 5:**
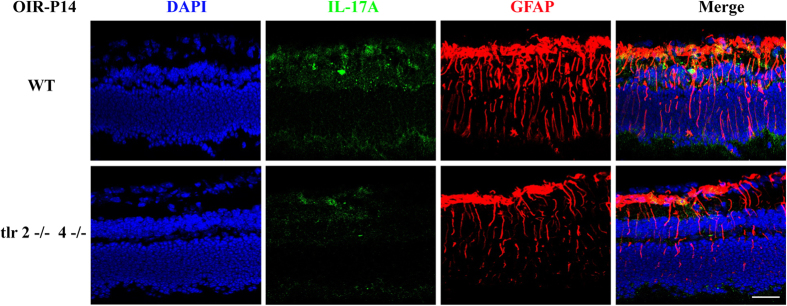
TLR2/4 deficiency suppresed IL-17A expression in retina. Immunofluorescence in cryosections revealed that the GFAP staining spread throughout the whole layers of the retina in WT-OIR mice, in contrast to the lower level of GFAP staining in TLR2/4 deficient mice. In addition, IL-17A is distributed dispersedly, especially in the GCL and ONL, and partly merged with GFAP staining in WT-OIR mice, On the contrary, TLR2/4 knockout mice showed little IL-17A staining in the retina, indicating that TLR2/4 improved IL-17A expression in retina. Scale bar: 50 μm.

To determine the IL-17A profile induced by TLR2/4 and its targeted cell in retinae, we used immunofluorescence with co-staining of IL-17A and GFAP in retinal cryosections. Müller cells from WT-OIR retinas showed evident activation and gliosis, with GFAP expression spread throughout the whole layers. Coincidently, IL-17A was distributed dispersedly and partly merged with GFAP staining (Fig. 5, upper panel). In the case of TLR2/4 KO mice, Müller cell gliosis was reduced markedly with less long endfeet GFAP expression across the retina, and less scattered IL-17A staining was observed in the GCL and ONL (Fig. 5, lower panel). We further cultured Müller cells *in vitro* and stimulated them with LPS, a classic ligand for both TLR2 and TLR4. The immunostaining revealed that LPS could activate the surface TLR2 and TLR4 of Müller cells (Fig. S1A,B), indicating the potential role of TLR2/4 signaling on biological effect of Müller cells. In addition, we and other studies found that the receptor for IL-17A (IL-17RA) could be expressed in glia cells *in vitro* (Fig. S2A) and *in vivo* (Fig. S2B), indicating that glial cells may be the targeted cells of the IL-17A cytokine^28^. Taken together, these data suggested that TLR2/4 deficiency suppressed the IL-17A expression in retina from OIR model.

**Figure 6:**
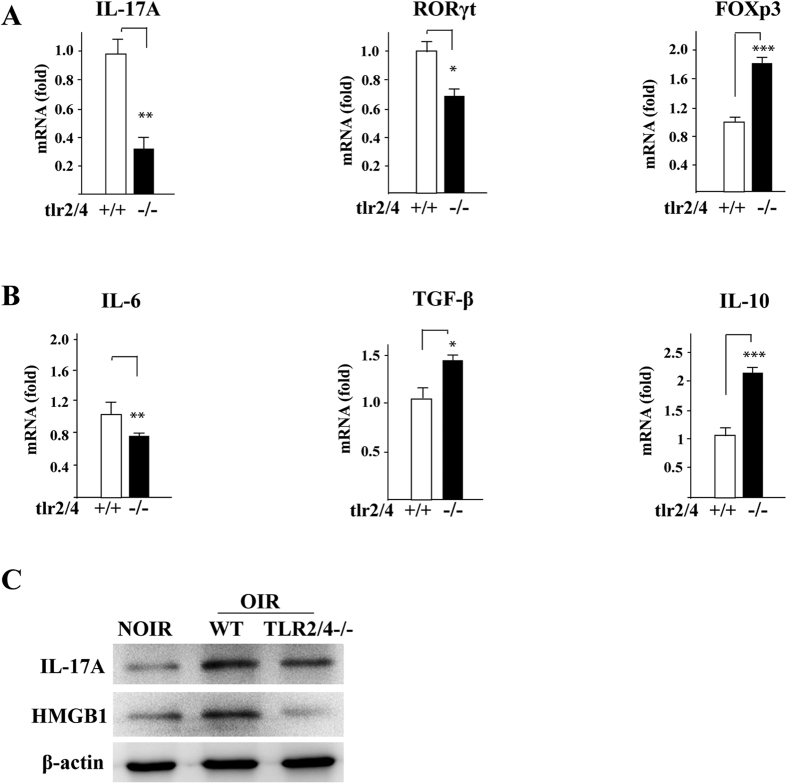
TLR2/4 promoted Th17 cells-related gene expression in the OIR model. **(A)** Real-time PCR results showed that the expression of IL-17A in the retina is reduced in TLR2/4 deficient mice compared to WT mice. RORγt, an important transcription factor for the differentiation of Th17 cells, was also decreased. On the other hand, Foxp3, the master transcription factor in Treg, had a marked increase in TLR2/4 KO mice. n = 10 **(B)** Low IL-6 expression, but increased TGF-β and IL-10 production in TLR2/4 deficient mice compared to WT mice. n = 10 **(C)** Western blot results revealed that the protein expressions of IL-17A and HMGB1 were markedly elevated in the WT-OIR retinae compared to WT-NOIR ones, however, their expression were much lower in TLR2/4−/− -OIR retinae than those in WT-OIR. *p < 0.05, **p < 0.01, ***p < 0.001.

To quantify the IL-17A expression in OIR *in vivo*, real-time PCR was used to determine the mRNA expression of IL-17A in retinae. As shown in Fig. 6A, TLR2/4 deficiency reduced IL-17A expression in the retina by more than three-fold compared with the WT-OIR model. The results of western blot further showed the protein expression of IL-17A was increased significantly in the WT-OIR retinae compared to WT-NOIR ones, while slightly increased IL-17A level was observed in TLR2/4−/− OIR mice (Fig. 6C). These data suggested that TLR2/4 may promote the expression of IL-17A in retina in the OIR model.

### TLR2/4 regulated IL-17A-related gene expression in the OIR model

To determine whether TLR2/4 regulates the expression of IL-17A-related genes, we next used real-time PCR to measure the mRNA levels of relevant transcription factors and cytokines in OIR retinae from WT and TLR2/4−/− mice. As shown in Fig. 6A, RORγt, an important transcription factor for the differentiation of Th17 cells, was reduced in the TLR2/4−/− group compared with WT mice. In contrast, Foxp3, the master transcription factor in Treg, had a marked increase in TLR2/4−/− mice. IL-6, TGF-β, and IL-10 are essential regulator cytokines for inducing or suppressing Th17 cell differentiation. Thus we also detected the expression of these cytokines and found that TLR2/4 deficiency resulted in a reduced IL-6 expression but an increased production of TGF-β and IL-10 (Fig. 6B). In addition, to evaluate the potential ligands for TLR2/4 activation in OIR to trigger IL-17 production, the expressions of HMGB1 and HSP60 in OIR retina from WT and TLR2/4 deficient mice were detected. The western blot results revealed the HMGB1 expression increased significantly in the WT-OIR retina compared with WT-NOIR ones while HMGB1 level in the TLR2/4 deficient mice was substantially reduced (Fig. 6C), indicating that HMGB1, recognized as a TLR4 ligand, activated TLR2/4, of which signaling might be involved in the IL-17 production in OIR model. No significant changes of HSP60 expression was observed between different groups (data not shown).

### IL-17A promotes the activation and proliferation of glial cells

**Figure 7:**
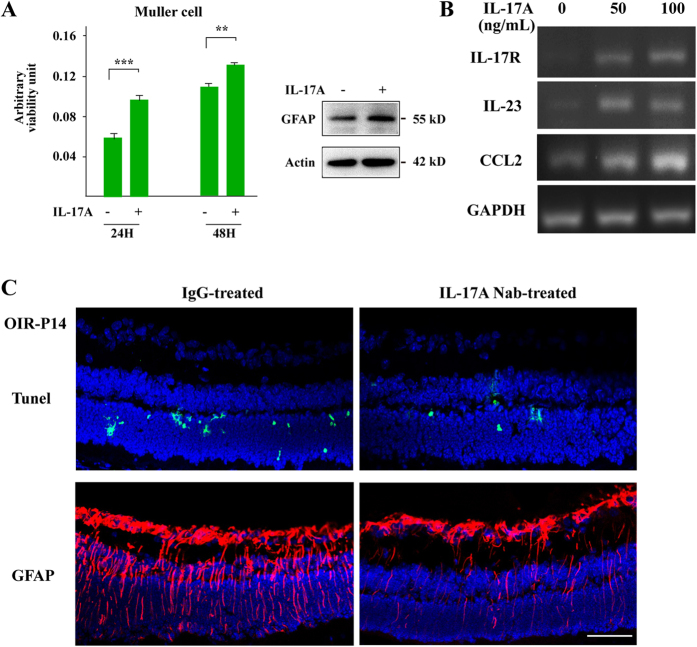
IL-17A promoted the activation and proliferation of glia cells. **(A)** A MTT assay revealed that IL-17A increased the proliferation of Müller cells after 24 hours or 48 hours. In addition, western blot assays showed that IL-17A promoted the protein expression of GFAP in Müller cells. **(B)** In immortalized BV-2 microglia cell, IL-17A stimulation increased the expression of IL-17R, IL-23 and CCL2. **(C)** Tunel staining results showed that IL-17A blockade reduced the Tunel-positive apoptotic cells in the OIR retina. In addition, GFAP-positive activated glial cells were significantly reduced after IL-17A neutralizing antibody treatment. Scale bar: 50 μm. n = 6, **p < 0.01, ***p < 0.001.

To further evaluate the role of IL-17A on retinal cells, we first isolated and stimulated the Müller cells with IL-17A cytokine. The MTT assay results revealed that IL-17A increased the proliferation of Müller cells (Fig. 7A). In addition, western blot analysis showed that overall GFAP protein expression was slightly upregulated in IL-17A-treated Müller cells, indicating that IL-17A contributes to the proliferation and activation of glial cells. In the context of another glia cell line, IL-17 could promote other pro-inflammatory genes expression such as IL-23 and CCL2 in BV-2 microglia cell (Fig. 7B), showing the potential role of IL-17 on activating microglia cells. Furthermore, we used a neutralizing antibody to evaluate the role of IL-17A on the apoptosis and activation of retinal cells in OIR mice. Tunel staining results showed that IL-17A blockade reduced cell apoptosis at P14 in the OIR retina (Fig. 7C, Upper panel; Fig. S3). In addition, there were significantly reduced GFAP-positive activated glial cells after IL-17A blockade (Fig. 7C, Lower panel), indicating that IL-17A contributes to the activation of glia cells in OIR mice. Taken together, these data suggested that IL-17A is required in activating glial cells during OIR.

## Discussion

TLR2 and TLR4 contributes to angiogenesis in the central nervous system^29^,^30^. In this study, we showed that TLR2/4 deficiency attenuated retinal vessel regression under hyperoxia conditions in an OIR model, revealing the important role of TLR2 and TLR4 in vascular degeneration. Simultaneously, retinal glial activation is alleviated in TLR2/4 deficient mice, accompanied with increased revascularization compared with WT mice. We further provided evidence that this effect is mediated by IL-17A-mediated inflammation. IL-17A blockade suppresses retinal cell apoptosis and glial cell activation. Thus, TLR2/4-mediated IL-17A signaling is required in retinal vascular degeneration, indicating that targeting TLR2/4-mediated IL-17A signaling is a novel strategy to promote revascularization and treat degenerative diseases.

The TLRs, especially TLR2 and TLR4, are expressed in the retina and could play an important role in inducing immune responses or regulating the inflammatory condition in retina^31^,^32^,^33^,^34^,^35^. As a bridge of innate and adaptive immune responses, TLR2 and TLR4 orchestrate retinal innate responses under several pathological condition, such as bacterial endophthalmitis and other infectious diseases^31^,^32^,^33^,^34^,^35^. TLR2 and TLR4 are expressed widely in various retina cells including Müller cells, retina pigment cells, microglia and astrocytes, made them possible critical mediators in the retinal diseases.

Simultaneous activation of different TLRs can exert synergistic effects and various ligands can activate the same specific TLR^22^,^23^. Both TLR2 and TLR4 are expressed and activated in immune cells, neural cells, and vascular cells, establishing TLR2 and TLR4 as a link between the immune response and neurovascular diseases. The potential synergistic role of TLR2 and TLR4 has been deeply investigated^24^,^25^. These receptors share ligands, such as high mobility group B1 (HMGB1) and exert similar roles in inflammation-related angiogenesis^6^,^29^. In this study, we also found HMGB1 is involved in TLR2/4-mediated retinal inflammation. Double knockout TLR2 and TLR4 may fully inhibit the TLR-related inflammatory response in the retinal vascular pathological condition. In this study, the TLR2/4 deficient mice showed a considerable alleviation of retinal vessel degeneration and glial activation compared with WT mice, suggesting that the activation of both TLR2 and TLR4 may accelerate vascular degeneration.

Retinal glia are activated in response to hypoxia to restore the oxygen supplement, however, this response can be harmful under consistent hypoxia, leading to impaired revascularization^26^. In normal retina, glial cells ensheathe the vasculature and establish neurovascular coupling, indicating the potential role to regulate vascular biology^36^. It is a general mechanism that glial cells react to an injury to protect the retinal tissue however this event induces subsequent secondary damage. In the present study, TLR2/4 deficiency attenuated the glial activation and promoted revascularization, suggesting that TLR2/4-mediated immune response enhanced the destructive glial activation. In addition, the excessive reactive glia in WT-OIR was accompanied with less revascularization compared with TLR2/4 knockout mice, indicating that TLR2/4 signaling is required in glia activation and impaired revascularization.

IL-17A is regarded as a novel member of the angiogenic factor family and has a prominent role in angiogenesis-related diseases^19^,^29^,^37^. The proinflammatory IL-17A cytokine was also increased in aged coronary arteries and atherosclerotic lesions, extending its role in vascular dysfunction and vascular diseases^38^,^39^. In this study, TLR2/4 deficiency increased the expression of IL-17A and its related genes in retinae from OIR mice, revealing the potential role of IL-17A in vessel regression and revascularization. In addition, Th17 cells produce IL-17A; however, other immune cells can also generate IL-17A, such as innate immune cells, NK cells, neutrophils, and even microglia^40^,^41^. Our and other studies revealed that The IL-17RA can be expressed by astrocytes or Müller cells, which are critical responders to IL-17A recognition in the central nervous system^41^,^42^. Furthermore, using a neutralizing antibody, we found that IL-17A blockade alleviated hypoxia-induced glial activation and retinal cell apoptosis, especially in the outer nuclear layer, which is rich in Müller cellular nuclei, indicating that IL-17A may mediate the activation and apoptosis of Müller cells *in vivo*. *In vitro*, IL-17A could also promote the proliferation and activation of Müller cells. Thus, these data suggested that IL-17A could mediate impaired retinal glial function in an OIR model.

Interestingly, the elevation of IL-17A-expressing cells in retina was coincident to the Th17-skewed cells in the spleen of WT-OIR mice compared with TLR2/4−/− OIR ones in this study, indicating that TLR2/4 signaling was involved in the generation, migration, and infiltration of IL-17A-expressing cells into the retinae, which mediated the retinal inflammation and vascular damage and repair. Although the eye is generally considered to be an immune privileged organ with a complete blood-retinal barrier, inflammation may damage the barrier and break this tolerance. The CD4^+^ T cells differentiation mostly occur in peripheral immune system and the differentiated CD4^+^ cell might be recruited into the local inflamed tissue and amplify the inflammation response^43^,^44^. Thus there is a possibility that the prominent Th17-skewed CD4 T cells in peripheral lymphoid organs may enter the retina and induce IL-17A-mediated inflammation in retina. In addition, TLR2/4 have been reported to play a pivotal role in T cell activation and polarization to the desired Th17 subset^45^,^46^ and enhance the production of IL-17A cytokine^47^,^48^, shaping the local and systemic immune responses. The Th17 subtype can aggravate angiogenesis in part through the secretion of IL-17A^49^. In contrast, IL-10-expressing Treg cell could regulate and suppress the TLR2/4-initiated inflammation, contributing to prevention of pathological angiogenesis. In this study, we found reduced Th17 cells and increased Treg cells in both retina and spleen from OIR model of TLR2/4−/− mice, indicating the involvement of TLR2/4 in Th17-skewed T cell polarization and trafficking in oxygen-induced vessel degeneration and revascularization.

In summary, TLR2/4-mediated inflammation orchestrated the immune response to vascular diseases. The present study made several new observations that TLR2/4 deficiency protected against retinal vessel degeneration and reduced retinal glia activation with more revascularization. In addition, TLR2/4 induced IL-17A inflammatory signaling and tipped the balance between Th17 cells and Tregs towards Th17 cells, uncovering a general immune mechanism of vessel degeneration. Thus, the TLR2/4-mediated IL-17A inflammation pathway is involved in vascular degeneration and revascularization, which suggests that this pathway may be a novel target for the treatment of vascular degenerative diseases.

## Methods

### Animals

TLR2/4 double-gene knockout (KO) mice were generated by backcross of TLR2 and TLR4 single KO mice, which were originally generated by Shizuo Akira (Osaka University, Osaka, Japan). Littermates of both sexes between 7 and 23 days old were used in all experiments. Animals were kept in a specific pathogen-free facility and maintained on an irradiated sterile diet and provided with clean water. The study protocol was reviewed and approved by the animal experimental ethics committee of Zhongshan Ophthalmic Center, Sun Yat-sen University, China (authorized number: 2013-085). The methods were carried out in accordance with the approved guidelines of Animal Care and Use Committee of Zhongshan Ophthalmic Center and the Association Research in Vision and Ophthalmology (ARVO) Statement for the Use of Animals in Ophthalmic and Vision Research.

### Mouse model of oxygen-induced retinopathy

The procedures to produce the oxygen-induced retinopathy model were based on the previously described method of Smith *et al*.^50^. On postnatal day (P) 7, mouse pups and the nursing mother were placed in an airtight incubator (own production) ventilated by a mixture of oxygen and air to a final oxygen fraction of 75% ± 2%. The oxygen levels were checked at least three times per day. After five days of hyperoxia, these mice were returned to room air at P12. The OIR model comprises two critical stages of vascular pathologic process. During the first phase of hyperoxic exposure (P7-P12), immature retinal vessels regressed and the development of the normal retinal vasculature was delayed, leading to a central zone of vaso-obliteration (VO). After returning mice to room air at P12, the central avascular retina becomes relative hypoxic, triggering both normal vessel regrowth, which predominantly occur within the VO with relative normal shape of vasculature before P14, and a pathologic formation of extraretinal neovascularization, which occurred at the border of VO and periphery retinal vasculature after P14 with the maximum severity at P17. In this study, the central avascular area was measured at P12 and P14. Some C57/B6 mice at P7 received a single intravitreal injection of IL-17 neutralizing antibody (2 μg/eye, R&D systems) and were then used to establish the OIR model.

### Vascular and glial immunofluorescence staining in retinal whole mounts

Vascular regression and revascularization were analyzed using immunofluorescence staining on retinal whole mounts. The perfusion of the retinal vessels with FITC-conjugated dextran was performed as described previously^6^. Briefly, P12 and P14 day-old mice were anesthetized and perfused via the left ventricle with 1 mL of 50 mg/mL FITC-conjugated dextran (Mw = 2 × 10^6^ Da; Sigma-Aldrich) in 0.15 M PBS. Then, the retinae were dissected, flatmounted, and viewed under a fluorescence microscope (Axioplan 2 imaging; Carl Zeiss). In addition, we also performed isolectin GS-B4 staining of the whole mounts. Briefly, the eyes were enucleated and fixed in 4% paraformaldehyde in 0.1 M PBS for 45 minutes. Retinae were dissected out as a cup and incubated with isolectin GS-B4 from Griffonia simplicifolia, Alexa Fluor 488 conjugate (1:200; Invitrogen) overnight at 4 degree. In addition, a primary antibody against glial fibrillary acidic protein (GFAP) (1:500, Dako) was used in the immunofluorescence staining of retinal whole mounts to detect retinal glia activation. Each retina was washed three times with PBS and mounted. Images were obtained using a Zeiss Axiophot fluorescent microscope and LSCM (LSM510, Carl Zeiss). The images were processed uniformly using Adobe Photoshop CS5. Quantitative analysis of the vasculature was carefully delineated based on pixel intensities, as previously described^6^. The area of the avascular region was calculated using ImageJ (National Institutes of Health) and shown as the percentage of the total retinal area. Revascularization was calculated as the difference of the avascular area from P12 to P14 divided by the avascular area at P12.

### Immunofluorescence staining in frozen section and cells slides

Eyes were enucleated and embedded in OCT compound (Tissue-Tek; Sakura Fine Technical, Torrance, CA) overnight for cryosectioning, and then, 8-μm serial sections were cut. Frozen sections of mouse eyes were dried at room temperature and postfixed in cold acetone for 10 minutes. The cells slides were washed with PBS twice and fixed in 4% PFA for 20 minutes. Sections or cells slides were washed with PBS, permeated with 0.5%Triton-X100/PBS for 5 minutes and blocked with 1% BSA in PBS for 1 hour at room temperature. Slides were incubated with rat anti-GFAP antibody (Dako) and rabbit anti- IL-17A antibody (Abcam) overnight at 4 °C. Then, the slides were rinsed three times with PBS/0.05% Tween-20 and incubated with donkey anti-rat IgG (H+L), Alexa Fluor^®^ 555, and donkey anti-rabbit IgG (H+L), Alexa Fluor^®^ 488, secondary antibodies (Invitrogen) at room temperature for 1 hour. The slides were thoroughly washed and counterstained with DAPI at 1:1,000 (Invitrogen) for 4 minutes at room temperature. After repeated washing with PBS/Tween-20, the slides were mounted and analyzed using the LSCM Imager and AxioVision software (Carl Zeiss). Apoptosis in the frozen sections was detected using a TUNEL Kit (Roche) following the manufacturer’s instructions. Fluorescence pictures were taken with identical exposure settings. For negative control, slides without primary antibody incubation showed no signals.

### Detection of IL-17A mRNA levels and related genes by real-time PCR

Real-time PCR analysis was performed to detect the mRNA level of IL-17A and related genes in the retinae. The total RNA of the retinae was extracted with TRIzol (Invitrogen, Carlsbad, CA, USA) and converted into first-strand cDNA using random hexamer primers and the Reverse Transcriptase Superscript II Kit (Invitrogen) according to the manufacturer’s instructions. PCR was then performed in a total volume of 20 μL containing 2 μL of cDNA, 10 μL of 2 × SYBR Premix Ex Taq, 0.8 μL of 50 × ROX Reference Dye (TaKaRa Biotechnology Co., Ltd, Dalian, China), and 10 μmol/L of the primer pairs. The primers are listed in Table S1. β-actin was used as a reference gene. The PCR amplification protocols consisted of 95 °C for 30 s and up to 40 cycles of 95 °C for 5 s and 60 °C for 34 s according to the manufacturer’s instructions. The inline image method was used to normalize the expression of the genes of interest relative to an internal control gene.

### Analysis of Th17 and Treg cells by flow cytometry

Cells were isolated from the retina, spleen or lymph nodes as previously described^51^. For intracellular cytokine detection, the cells were re-stimulated for 5 hours with PMA (20 ng/ml; Sigma-Aldrich)/ionomycin (1 μM; Sigma-Aldrich). Brefeldin A (1 μl/ml; BD Biosciences) was added in the last two hours, and staining for intracellular cytokine was performed. Briefly, cells were stained on ice for 30 min with a surface monoclonal antibody, and then fixed in 2% paraformaldehyde A for 10 minutes. After permeation with 0.1% Triton-X in 5% BSA, the cells were incubated with intracellular cytokine antibody. A four-laser Becton-Dickinson FACSCalibur (BD Biosciences) was used to collect the data, and FlowJo software was used for analysis.

### Retinal glia cells culture and MTT assay

Primary Müller cells were isolated and cultured. In brief, the retinae of C57BL/6 pups at postnatal 4–7 days were isolated, chopped and cultured in DMEM/F12 (1:1 ratio of Dulbecco’s Modified Eagle’s Medium and Ham’s F12 medium) with 10% fetal bovine serum (FBS) (Invitrogen, California, US) in a humidified environment of 5% (vol./vol.) CO_2_ at 37 °C. After 3 days, the retinal aggregates were vigorously rinsed and the cells were kept in and fed with medium. The cells that were validated as being Müller cells by their positive glutamine synthetase immunocytochemical staining were used for further experiments. The immortalized murine microglial cell line BV-2 was cultured in RPMI 1640 medium supplemented with 5% FBS. The retinal glia cells were stimulated with 100 ng/mL of recombinant IL-17A after serum-starvation overnight. Twenty-four hours later, the cellular protein was collected and used for detecting GFAP expression by western blot assays, and cell proliferation was evaluated in a MTT assay (MP Biomedicals, California, US) according to the manufacturer’s instruction.

### Statistical analysis

Data are presented as the mean ± standard error of the mean (SEM). Differences between two groups were analyzed using the independent t-test. The significance levels are marked *P < 0.05; **P < 0.01; ***P < 0.001.

## Additional Information

**How to cite this article**: He, C. *et al*. TLR2/4 deficiency prevents oxygen-induced vascular degeneration and promotes revascularization by downregulating IL-17 in the retina. *Sci. Rep.* **6**, 27739; doi: 10.1038/srep27739 (2016).

## Supplementary Material

Supplementary Information
