# Supplementary material for: TLR2/4 deficiency prevents oxygen-induced vascular degeneration and promotes revascularization by downregulating IL-17 in the retina
Source: Sci Rep. 2016 Jun 14;6:27739. doi: 10.1038/srep27739 (PMC4906284; doi:10.1038/srep27739)
Supplement: Supplementary Information [file srep27739-s1.pdf]

TLR2/4 deficiency prevents oxygen-induced vascular degeneration and promotes revascularization by downregulating IL-17 in the retina

Chang He[1#], Peilong Lai[2#], Jing Wang[1], Tian Zhou[1], Zijing Huang[1], Lingli Zhou[1], Xialin Liu[1*]

[1]State Key Laboratory of Ophthalmology, Zhongshan Ophthalmic Center, Sun Yat-Sen University, Guangzhou 510060, P. R. China;

[2]Department of Haematology, Guangdong General Hospital, Guangdong Academy of Medical Sciences, Guangzhou 510080, P. R. China;

# These authors contributed equally to this work.

*Corresponding author: Xialin Liu (liuxl28@mail.sysu.edu.cn)

State Key Laboratory of Ophthalmology, Zhongshan Ophthalmic Center,

Sun Yat-Sen University, 54 South Xianlie Road, Guangzhou 510060

Guangdong, P. R. China, Tel & fax: 0086-20-87330294

Supplementary information

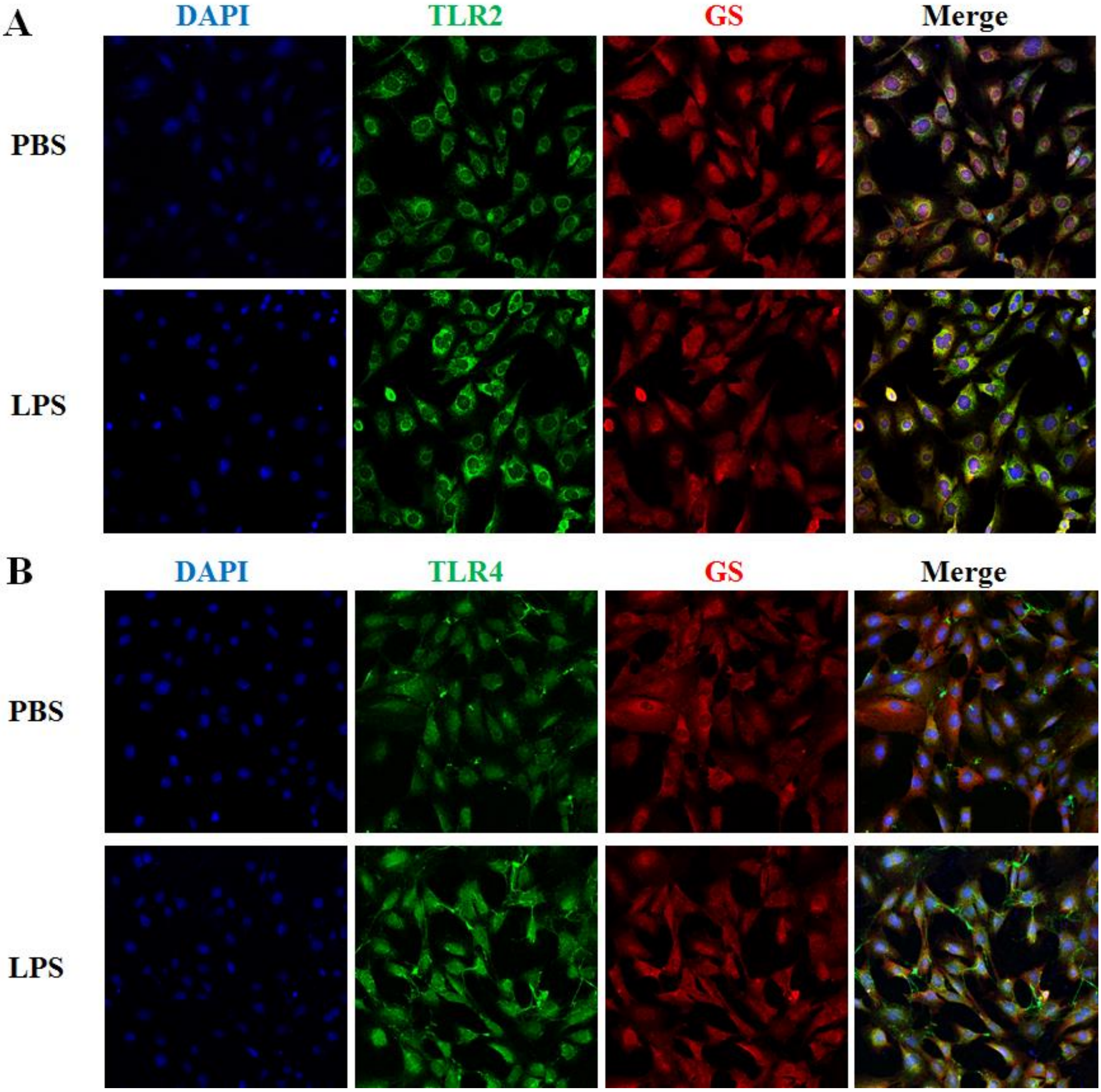

Figure S1. TLR2 and TLR4 activation in response to LPS stimulation in retinal Müller cells.

A dose of 100 ng/mL LPS could significantly activate the TLR2 **(A)** and TLR4 **(B)** on cultured retinal Müller cells.

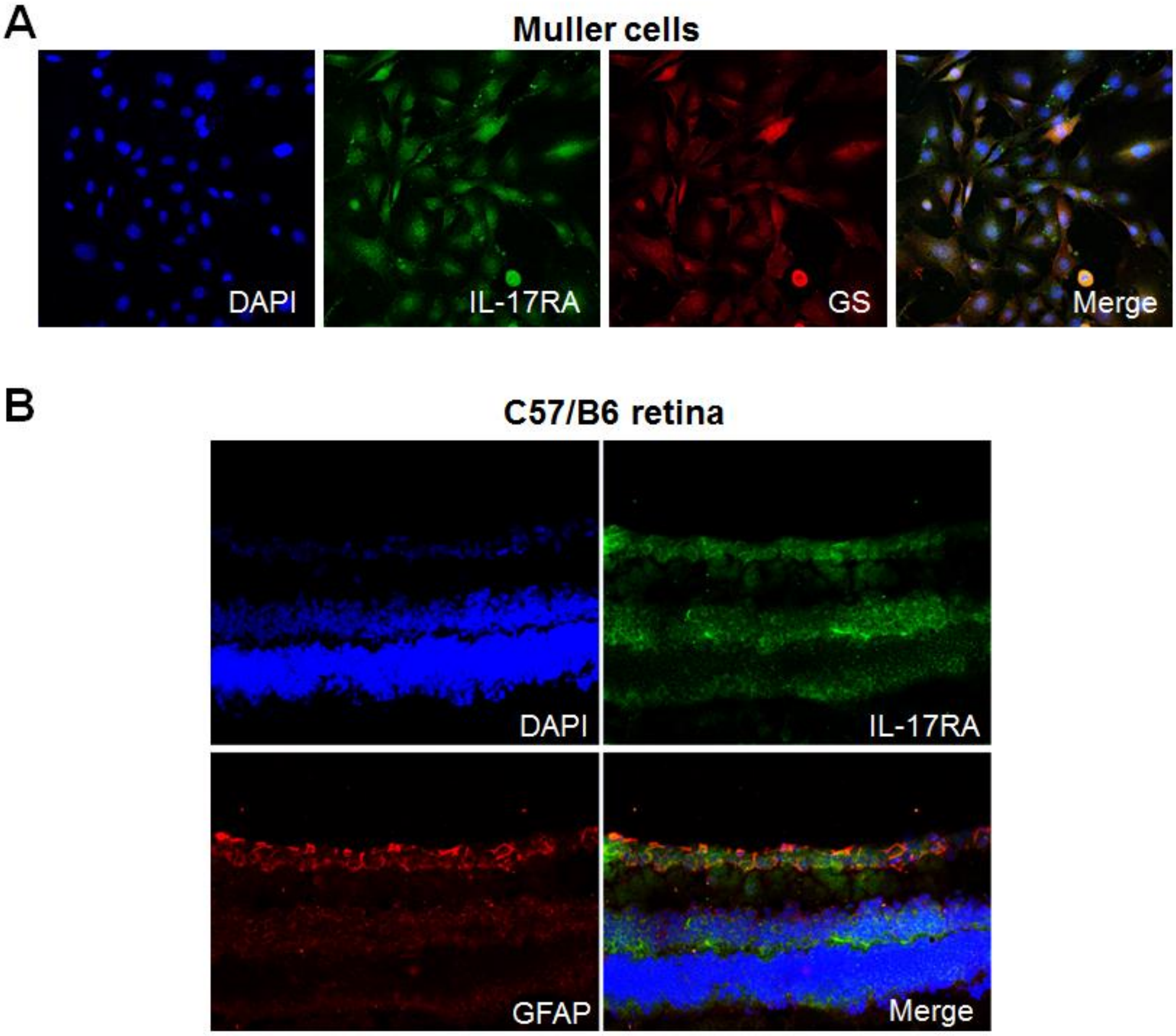

Figure S2. Receptor for IL-17A is abundantly expressed on retinal glia cells.

(A) The receptor for IL-17A (IL-17RA) is highly expressed in cultured Müller cells, which are identified by specific GS marker. **(B)** In C57/B6 mice, the IL-17RA is mainly distributed in GCL and INL in retina. Importantly, part of IL-17RA positive cells is co-staining with GFAP, glial cells activation marker in retina.

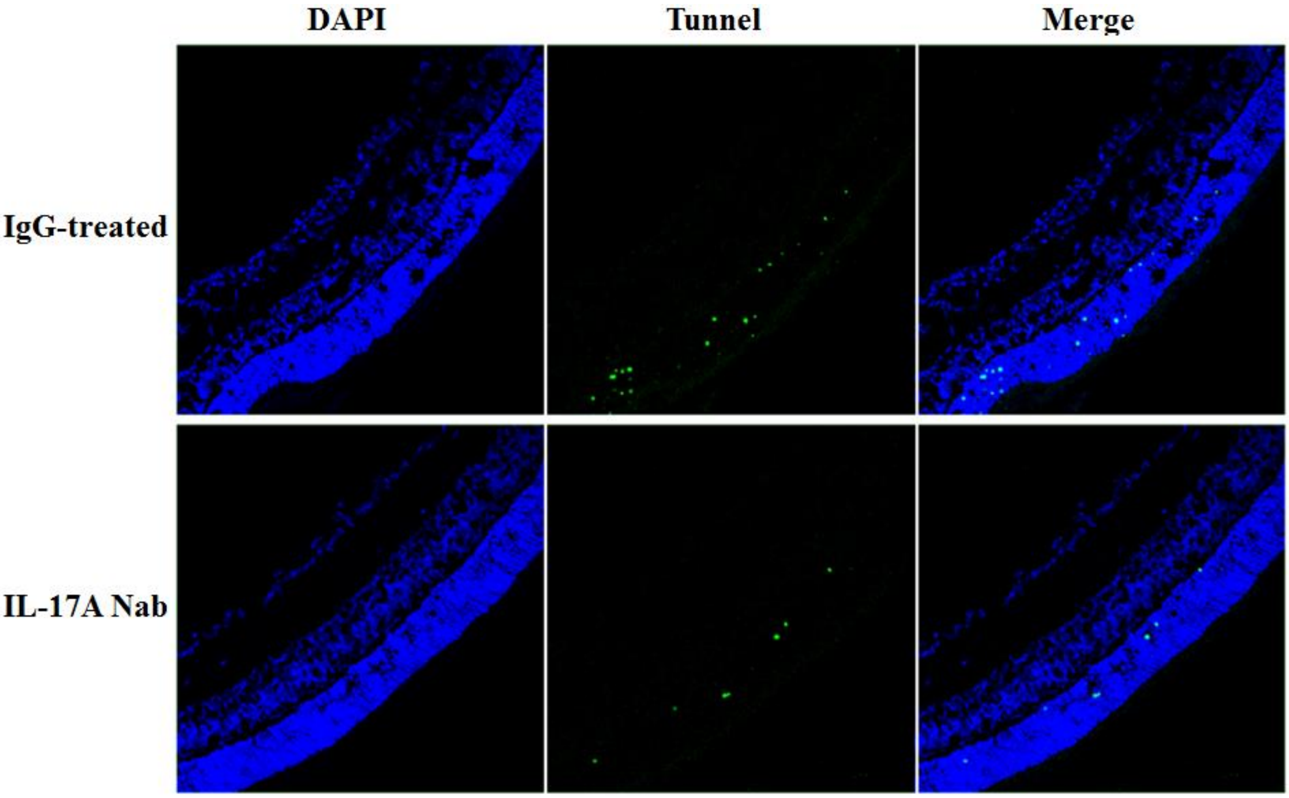

Figure S3. Low magnification images of Tunel staining after IL-17A blockade.

The confocal images under (200X) were captured to show larger field of retinal sections. IL-17A blockade using neutralizing antibody suppressed retinal cellular apoptosis, presented as less Tunel-positive cells in the OIR retina.

Table S1. Primers used for real-time PCR

Gene name	Orientation	Primer sequence (5' to 3')	Species
tgf-β	Forward	TGCACCCAAACCGAAGTCAT	Mouse
	Reverse	TTGTCAGAAGCCAGCGTTCAC	Mouse
il-6	Forward	CAAAGCCAGAGTCCTTCAGA	Mouse
	Reverse	GATGGTCTTGGTCCTTAGCC	Mouse
rorrt	Forward	CCGCTGAGAGGGCTTCAC	Mouse
	Reverse	TGCAG-GAGTAGGCCACATTACA	Mouse
il-10	Forward	CCCTTTGCTATGGTGTCCTT	Mouse
	Reverse	TGGTTTCTCTTCCCAAGACC	Mouse
il-17	Forward	GGTCAACCTCAAAGTCTTTAACTC	Mouse
	Reverse	TTAAAAATGCAAGTAAGTTTGCTG	Mouse
foxp3	Forward	CCCAGGAAAGACAGCAACCTT	Mouse
	Reverse	TTCTCACAACCAGGCCACTTG	Mouse
gapdh	Forward	TGAGCAAGAGAGGCCCTATC	Mouse
	Reverse	AGGCCCCTCCTGTTATTATG	Mouse
